# Selective ^1^H-^14^N Distance Measurements by ^14^N Overtone Solid-State NMR Spectroscopy at Fast MAS

**DOI:** 10.3389/fmolb.2021.645347

**Published:** 2021-04-08

**Authors:** Nghia Tuan Duong, Zhehong Gan, Yusuke Nishiyama

**Affiliations:** ^1^ NMR Science and Development Division, RIKEN SPring-8 Center, Nano-Crystallography Unit, RIKEN-JEOL Collaboration Center, Yokohama, Japan; ^2^ Centre of Interdisciplinary Magnetic Resonance, National High Magnetic Field Laboratory, Tallahassee, FL, United States; ^3^ JEOL RESONANCE Inc., Tokyo, Japan

**Keywords:** ^1^H-^14^N distances, ^14^N overtone spectroscopy, PM-S-RESPDOR, REDOR, fast MAS frequency

## Abstract

Accurate distance measurements between proton and nitrogen can provide detailed information on the structures and dynamics of various molecules. The combination of broadband phase-modulated (PM) pulse and rotational-echo saturation-pulse double-resonance (RESPDOR) sequence at fast magic-angle spinning (MAS) has enabled the measurement of multiple ^1^H-^14^N distances with high accuracy. However, complications may arise when applying this sequence to systems with multiple inequivalent ^14^N nuclei, especially a single ^1^H sitting close to multiple ^14^N atoms. Due to its broadband characteristics, the PM pulse saturates all ^14^N atoms; hence, the single ^1^H simultaneously experiences the RESPDOR effect from multiple ^1^H-^14^N couplings. Consequently, no reliable H-N distances are obtained. To overcome the problem, selective ^14^N saturation is desired, but it is difficult because ^14^N is an integer quadrupolar nucleus. Alternatively, ^14^N overtone (OT) NMR spectroscopy can be employed owing to its narrow bandwidth for selectivity. Moreover, owing to the sole presence of two energy levels (*m* = ± 1), the ^14^N OT spin dynamics behaves similarly to that of spin-1/2. This allows the interchangeability between RESPDOR and rotational-echo double-resonance (REDOR) since their principles are the same except the degree of ^14^N OT population transfer; saturation for the former whereas inversion for the latter. As the ideal saturation/inversion is impractical due to the slow and orientation-dependent effective nutation of ^14^N OT, the working condition is usually an intermediate between REDOR and RESPDOR. The degree of ^14^N OT population transfer can be determined from the results of protons with short distances to ^14^N and then can be used to obtain long-distance determination of other protons to the same ^14^N site. Herein, we combine the ^14^N OT and REDOR/RESPDOR to explore the feasibility of selective ^1^H-^14^N distance measurements. Experimental demonstrations on simple biological compounds of L-tyrosine.HCl, N-acetyl-L-alanine, and L-alanyl-L-alanine were performed at 14.1 T and MAS frequency of 62.5 kHz. The former two consist of a single ^14^N site, whereas the latter consists of two ^14^N sites. The experimental optimizations and reliable fittings by the universal curves are described. The extracted ^1^H-^14^N distances by OT-REDOR are in good agreement with those determined by PM-RESPDOR and diffraction techniques.

## Introduction

H-N distance is of importance for deeper insights into the structures and dynamics of chemical and biological systems due to the ubiquity of both proton and nitrogen. Such distance can be obtained by solid-state nuclear magnetic resonance (ssNMR) through the determination of H-N dipolar coupling, which is inversely proportional to the cube of the H-N distance. There are a few reasons why ssNMR has advantages for the H-N measurement over traditional diffraction techniques. First, ssNMR spectroscopy is applicable to various systems no matter their states, i.e., lacking long-range order or even being a disorder, which are intractable by diffraction techniques. Second, it enables the precise location of the H-atom positions, which is poorly determined by X-ray diffraction (XRD) or electron diffraction (Guzmán-Afonso et al., 2019).

Despite such advantages, the H-N distance measurement by NMR has difficulties due to some unfavorable nuclear characteristics of these two elements. For nitrogen, it has two stable and NMR active isotopes, ^15^N and ^14^N. The former is preferred in ssNMR because it is a spin-1/2 nucleus; thus, it is easy to manipulate and to obtains high resolution. Many methods have been designed to measure ^1^H-^15^N distances (Hohwy et al., 2000; Zhao et al., 2001; Schnell and Saalwächter, 2002; Fu, 2003; Chevelkov et al., 2009; Hou et al., 2011; Schanda et al., 2011; Paluch et al., 2013; Hou et al., 2014; Nishiyama et al., 2016). Nevertheless, the main drawback of ^15^N isotope is its insensitivity owing to the low natural abundance (0.4%). It makes the measurements lengthy for sufficient signal-to-noise ratio (S/N); otherwise, 1) the isotopic labeling is needed, which is not always simple and cost-effective or 2) dynamic nuclear polarization experiments are required (Zhao et al., 2018). However, the ^1^H-^15^N experiments on that work only allowed the determination of the shortest ^1^H-^15^N distance due to the dipolar truncation effect. On the other hand, ^14^N isotope benefits from the high natural abundance (99.6%), but it suffers from the severe quadrupolar broadening and complicated spin dynamics because ^14^N is an integer quadrupolar nucleus (spin *I* = 1). For protons, the intense ^1^H–^1^H homonuclear dipolar couplings in the solid state cause ^1^H line broadening and shorten the ^1^H coherence time. Consequently, these unfavorable characteristics of both N isotopes and H nucleus make H-N distance measurement by NMR challenging.

The development of fast magic-angle spinning (MAS, *ν*_R_ ≥ 60 kHz) with proton detection has made ^14^N NMR spectroscopy a routinely used method, overcoming the difficulty associated with quadrupolar interaction (Cavadini et al., 2006; Gan et al., 2007; Cavadini, 2010; Nishiyama et al., 2011; Brown, 2014; Pandey and Nishiyama, 2015; Shen et al., 2015; Pandey et al., 2016; Carnevale et al., 2017; Hung et al., 2019; Jarvis et al., 2019; Rankin et al., 2019; Wijesekara et al., 2020). Furthermore, under fast MAS conditions, the strong ^1^H–^1^H dipolar network is largely suppressed (Nishiyama, 2016). These two advantages potentially facilitate the ^1^H-^14^N distance measurement. Recently, our group have introduced a combination of phase-modulated (PM) pulse (Nimerovsky et al., 2014; Makrinich et al., 2017; Makrinich et al., 2018), SR4^2^_1_ recoupling (Brinkmann and Kentgens, 2006), and rotational-echo saturation-pulse double-resonance (RESPDOR) (Gan, 2006; Chen et al., 2010a; Chen et al., 2010b; Lu et al., 2011) (PM-S-RESPDOR) that can extract multiple ^1^H-^14^N distances with high accuracy at fast MAS of 70 kHz (Duong et al., 2019). Such success mainly comes from the robustness that universal fraction curves can be obtained for the distance measurement under the saturation by the PM pulse for a wide range of ^14^N quadrupolar coupling constant (*C*_Q_) and ^1^H-^14^N dipolar coupling. This broadband characteristics of PM pulse is useful when we work on systems containing a single ^14^N site, as shown in the previous study. However, complications may arise for systems where multiple ^14^N sites are present, as shown below.

**FIGURE 1:**
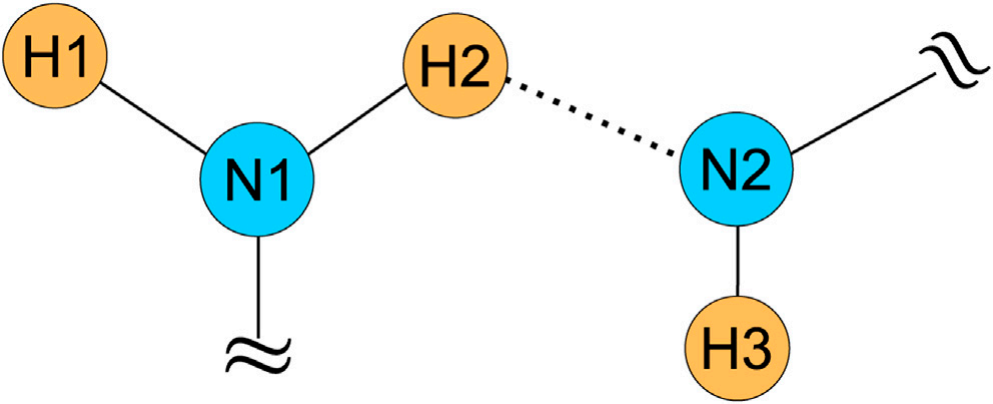
The 5-spin system with two N and three H atoms complicating the ^1^H-^14^N distance measurements by PM-S-RESPDOR.

For example, a 5-spin system, as shown in Figure 1, consists of two N and three H atoms. We assume that the ^14^N and ^1^H NMR peaks are well resolved for simplicity. The first difficulty associated with this system is the ambiguity of ^1^H-^14^N distance measurement. For instance, we can determine the distance of H3-N by PM-S-RESPDOR sequence but cannot know whether such distance is between H3 and N1 or H3 and N2. The second difficulty relates to the complex spin dynamics of H2 nucleus, which is close to both N1 and N2 nuclei. As PM pulse is broadband, it completely saturates both ^14^N1 and ^14^N2 nuclei; thus, the PM-S-RESPDOR sequence will give the H2-N fraction curve experiencing the combined effects of H2-N1 and H2-N2 pairs. The H2-N distance from the fraction curve would be shorter than those extracted from H2-N1 or H2-N2 pair; or in other words, no reliable distance is yielded. A solution to overcome this cumulative contribution is to selectively saturate each N nucleus, which can be achieved in the manner of Delays Alternating with Nutation for Tailored Excitation (DANTE) (Vitzthum et al., 2011; Vitzthum et al., 2012; Lu et al., 2013; Pourpoint et al., 2014). This approach can be our future work.

An alternative approach is the ^14^N overtone (OT) NMR spectroscopy, where the forbidden transitions |Δ*m*| = 2 are weakly allowed (*m* is the energy level) (Bloom and LeGros, 1986; Tycko and Opella, 1987; Jayanthi and Ramanathan, 2011; O’Dell and Ratcliffe, 2011; Nishiyama et al., 2013; O’Dell and Brinkmann, 2013; O’Dell et al., 2013; Haies et al., 2015a; Haies et al., 2015b; Shen et al., 2017; Concistré et al., 2018; Gan et al., 2018; Pandey and Nishiyama, 2018). Because it is twice the fundamental frequency, ^14^N OT frequency is more available to commercial MAS probes since many probes are not designed to tune to ^14^N Larmor frequency. Importantly, the ^14^N overtone excitation can achieve band-selective observation of ^14^N (Pandey and Nishiyama, 2018). The narrow bandwidth results from the slow effective ^14^N OT nutation, which is proportional to *C*_Q_/*ν*_0_, where *ν*_0_ is the ^14^N Larmor frequency. Besides the selectivity, ^14^N OT spectra are free from the first-order quadrupolar interaction because of the symmetric transitions, *m* = −1 ↔ *m* = +1. Therefore, ^14^N OT NMR is much narrower than the single-quantum ^14^N spectra and robust to the misadjustment of the magic angle. Moreover, since the transitions are only between two energy levels involved in OT (*m* = ±1), the spin dynamics of ^14^N OT behaves similarly to that of spin-1/2. Hence, for a ^1^H-^14^N OT system, the working conditions under RESPDOR can also be described by rotational-echo double-resonance (REDOR) (Gullion and Schaefer, 1989; Gullion, 2007) depending on whether the population transfer is saturation (RESPDOR) or inversion (REDOR) (Nimerovsky et al., 2017). Since the ideal saturation or inversion by continuous-wave (CW) is impractical, the working condition is an intermediate between REDOR and RESPDOR regimes. In this work, we combine ^14^N OT and REDOR sequence (^1^H-^14^N OT-REDOR) to explore its feasibility for distance measurements. This sequence is firstly demonstrated using two model biological compounds of L-tyrosine.HCl (Tyr) and N-acetyl-L-alanine (AcAla) and then applied to a more complex dipeptide system of L-alanyl-L-alanine (AlaAla) that involves two inequivalent nitrogen sites in a single molecule.

## Pulse Sequence and the Universal Expression

**FIGURE 2:**
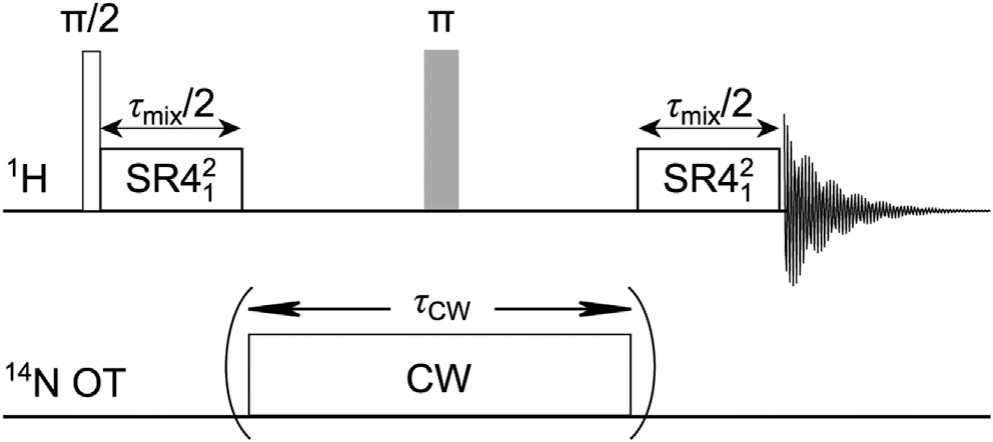
^1^H-^14^N OT-REDOR sequence. On ^1^H channel, SR4^2^_1_ recoupling is used to reintroduce the ^1^H-^14^N dipolar couplings. On ^14^N OT channel, CW pulse is used to perturb ^14^N OT populations. For the fraction curve, two sets of data (*S*_0_ and *S*′) are acquired by employing OT-REDOR sequence without and with CW pulse (within the two brackets), respectively.

Figure 2 depicts the ^1^H-^14^N OT-REDOR sequence. It is identical to the conventional S-REDOR sequence (Chen et al., 2010b), where SR4^2^_1_ recoupling (lasting for *τ*_mix_) is used to recover the ^1^H-^14^N dipolar coupling and CW (lasting for *τ*_CW_) is used to saturate/invert the ^14^N OT populations between the two energy levels. We note that since SR4^2^_1_ is not *γ*-encoded, the interval between the two SR4 blocks should be rotor-synchronized to avoid the spatial modulation of the recoupled ^1^H-^14^N dipolar couplings. For distance measurement, we measure two signals, *S*_0_ and *S*’, acquired without and with CW pulse, respectively, for obtaining the fraction curve Δ*S*/*S*_0_ = (*S*_0_–*S′*)/*S*_0_ as a function of *τ*_mix_.

The fraction curve excludes the signal attenuation from *T*_2_ relaxation, making it dependent solely on the dipolar coupling constant as for the case of REDOR with complete inversion. However, for ^14^N OT, complete inversion is difficult to achieve. Subsequently, the distance is extracted by fitting the fraction curve to either the numerically exact or universal curves. For the numerically exact curve, the simulation is extremely difficult as it must work in the laboratory frame without high-field approximation, leading to time-consuming calculations (O’Dell and Brinkmann, 2013). Even if this condition is met, various parameters must be known; for instance, the ^14^N *C*_Q_ and the ^14^N OT effective nutation fields, which are not straightforwardly determined. For the universal curve, it has shown to be an almost identical match to the numerically exact ^1^H-^14^N PM-S-RESPDOR curve, allowing simple distance extractions (Duong et al., 2019). In addition, the condition and knowledge required by the numerically exact ^1^H-^14^N OT-REDOR curve above are not necessary for the universal curve. Indeed, semiquantitative evaluation for ^14^N OT transitions only requires the fitting parameter *f* and the ^1^H-^14^N dipolar coupling (shown below). Thus, for objective fitting, we use the universal curve approach, which is derived by following the original work of Gan or later analysis of Chen and coworkers (Gan, 2006; Chen et al., 2010a). The derivation starts with fundamental ^14^N transitions for verification and then applies to ^14^N OT.

**FIGURE 3:**
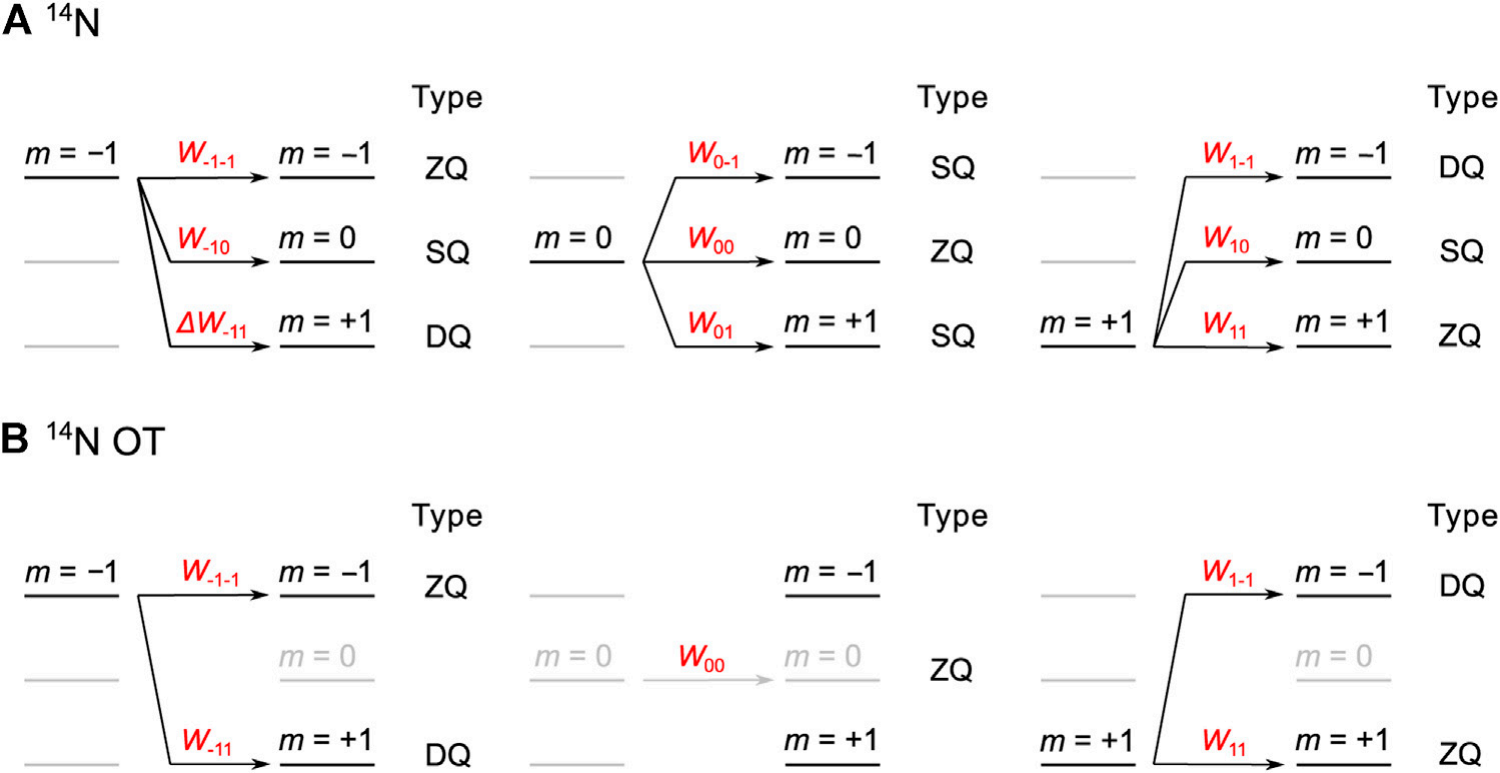
The population transfers and their probabilities (*W*_*ij*_) for ZQ (Δ*m* = 0), SQ (|Δ*m*| = 1), and DQ (|Δ*m*| = 2) transitions of **(A)** ^14^N and **(B)** ^14^N OT to derive the universal curves.

A general expression for any spin and type of experiment is

$$\frac{\Delta S}{S_{0}}=1-\underset{i,j}{\sum }P_{i}W_{ij}REDOR\left(\left|\Delta m\right|\right),$$ (1)

where *P*_*i*_ = 1/(2*I* + 1) is the population of spin state *m* = *i* under high-temperature approximation, *W*_*ij*_ is the population transfer probability from *m* = *i* to *m* = *j* spin state, and *REDOR*(|Δ*m*|) presents the normalized dipolar-dephased signal intensity for classical REDOR. The general expression helps to derive the universal curves mentioned in Figure 3. It is worth noting that the natural abundance of a specific isotope should also be considered in Eq. (1). However, the natural abundance of ^14^N isotope is 99.6%, very close to 100%; hence, we can safely neglect it.

For ^14^N (*I* = 1) spin, under the Zeeman interaction with the external magnetic field, there are three energy levels of *m* = 0 and ±1. We assume the population for each level is 1/3. The population transfers among the energy levels are categorized into zero- (ZQ or Δ*m* = 0), single- (SQ or |Δ*m*| = 1), and double-quantum (DQ or |Δ*m*| = 2) transitions, which determine the RESPDOR effect. Both energy levels *m* = +1 and *m* = −1 are involved in all three ZQ, SQ, and DQ transitions, as shown in Figure 3A. Under the ideal saturation of ^14^N spin, *W*_±1*j*_ for these transfers are equal; hence, each transition has *W*_±1*j*_ of 1/3. On the other hand, the energy level *m* = 0 is only involved in ZQ and SQ transitions, but there are two SQ transitions of (*m* = 0 → *m* = −1) and (*m* = 0 → *m* = +1). Hence, for *m* = 0, *W*_0*j*_ of ZQ, SQ, and DQ transitions are 1/3, 2/3, and 0/3, respectively. Taken together, under the complete saturation of ^14^N spin, Σ*P*_*i*_ *W*_*ij*_ for ZQ, SQ, and DQ transitions for *m* = (−1, 0, 1) are 1/3·3/3, 1/3·4/3, and 1/3·2/3, respectively (see Figure 3A). Replacing these *P*_*i*_ and *W*_*ij*_ in Eq. (1), the universal expression for ^14^N is given by

$$\begin{array}{l} \frac{\Delta S}{S_{0}}=1-\frac{3}{9}-\frac{4}{9}.REDOR\left(\left|\Delta m\right|=1\right)-\frac{2}{9}.REDOR\left(\left|\Delta m\right|=2\right)\;\;\;\;\;\;\;\;\;\;\;\;\;\;\;\;\;\\ =\frac{2}{3}-\frac{\pi \sqrt{2}}{9}J_{1/4}\left(\frac{\pi }{4}\left(b_{1H-14N}/2\pi \right)\tau_{mix}\right)J_{-1/4}\left(\frac{\pi }{4}\left(b_{1H-14N}/2\pi \right)\tau_{mix}\right)\\ -\frac{\pi \sqrt{2}}{18}J_{1/4}\left(\frac{2\pi }{4}\left(b_{1H-14N}/2\pi \right)\tau_{mix}\right)J_{-1/4}\left(\frac{2\pi }{4}\left(b_{1H-14N}/2\pi \right)\tau_{mix}\right), \end{array}$$ (2)

where *J*_±1/4_ denotes the ±1/4-order Bessel functions of the first kind and *b*_1H–14N_/(2π) is the ^1^H-^14^N dipolar coupling constant while *τ*_mix_ is the total mixing time of SR4^2^_1_ recoupling sequence. Eq. (2) is identical to the universal curve for ^1^H-^14^N RESPDOR in the literature (Gan, 2006; Chen et al., 2010b), verifying our analysis.

Next, we consider the case of ^14^N OT. Again, three energy levels are present with the population *P*_*i*_ of 1/3 for each level. The energy level *m* = 0 is not involved in OT transitions; thus, it is blurred in Figure 3B. *P*_*i*_ of *m* = 0 remains at ZQ transition, meaning that *W*_0*j*_ is 1 for *j* = 0 and 0 for *j* ≠ 0. Conversely, both energy levels *m* = ±1 are involved in the saturation/inversion of ZQ and DQ transitions. However, owing to the slow and orientation-dependent effective nutation of ^14^N OT, the complete saturation/inversion is difficult. Considering this incompletion, we assume that the DQ *W*_±1*j*_ for *m* = ±1 are *f* with 0 ≤ *f* ≤ 1, in which *f* = 0.5 corresponds to complete saturation while *f* = 1.0 corresponds to complete inversion. Although we mentioned that the working condition for ^1^H-^14^N OT-REDOR is between REDOR and RESPDOR regimes in the *Introduction* section, this does not mean that *f* should be between 0.5 and 1.0. Indeed, if the complete saturation is not achieved, parameter *f* could be smaller than 0.5. With the introduction of *f*, *W*_±1*j*_ for ZQ and DQ transitions are 1–*f* and *f*, respectively. Combining *P*_*i*_ of each transition for each level and under incomplete saturation/inversion of ^14^N OT, Figure 3B shows that Σ*P*_*i*_ *W*_*ij*_ for ZQ, SQ, and DQ transitions are 1/3·(1 + 2(1–*f*)), 0, and 1/3·2*f*, respectively. The universal expression for REDOR/RESPDOR on ^14^N OT is given by

$$\begin{array}{l} \frac{\Delta S}{S_{0}}=1-\left(1-\frac{2f}{3}\right)-\frac{2f}{3}.REDOR\left(\left|\Delta m\right|=2\right)\;\;\;\;\;\;\;\;\;\;\;\;\;\;\;\;\;\;\;\;\;\;\;\;\;\;\;\;\;\;\;\;\;\;\;\;\;\;\;\;\;\;\;\;\;\;\;\;\;\\ \;=\frac{2f}{3}\left[1-REDOR\left(\left|\Delta m\right|=2\right)\right]\\ =\frac{2f}{3}\left[1-\frac{\pi \sqrt{2}}{4}J_{1/4}\left(\frac{2\pi }{4}\left(b_{1H-14N}/2\pi \right)\tau_{mix}\right)J_{-1/4}\left(\frac{2\pi }{4}\left(b_{1H-14N}/2\pi \right)\tau_{mix}\right)\right]. \end{array}$$ (3)

From Eq. (3), the coefficient for REDOR (|Δ*m*| = 2) is proportional to *f*, affecting the slope of the fraction curve. However, since *f* uniformly affects the other elements in the equation, the universal curves derived from Eq. (3) would reach the maximum at the same *τ*_*mix*_ no matter *f* value. It is worth noting that the introduction of *f* makes the fitting among universal curves and experimental fraction curve better, but it makes the extracted *b*_1H–14N_ inaccurate. Particularly, when the fraction curve has not reached the maximum Δ*S*/*S*_0_, universal curves generated by different combinations of *f* and *b*_1H–14N_ can reproduce the very similar fraction curve, thus giving ambiguous results. To avoid this situation, our fitting strategy consists of two steps. The first is to determine *f*, which is possible only when the fraction curve of the shortest H-N distance must show the maximum. Under this condition, the fitting parameter *f* is determined as the ratio of the experimental and theoretical maxima Δ*S*/*S*_0_ (2/3 = 0.67). That precisely known *f* leads to the unambiguous determination of *b*_1H–14N_. For longer H-N distance, the REDOR curve may possibly not show the maximum while the oscillation is damped, making the fitting difficult. Under this situation, accurate distance determinations are still possible by the second step. It is to use this observed *f* from the first step for measuring longer ^1^H-^14^N distances of the same ^14^N site. This strategy is a disadvantage of OT-REDOR compared to PM-S-RESPDOR. The latter does not require the prior knowledge of the fitting parameter *f* owing to the complete saturation of all ^14^N crystallites by the PM pulse, thus enabling the reliable fitting even when the maximum is not observed.

**FIGURE 4:**
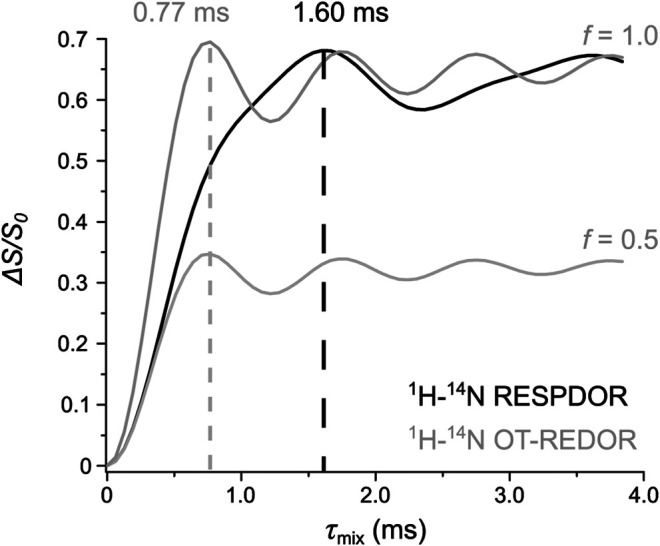
Comparison between the universal curves of ^1^H-^14^N RESPDOR (black) and ^1^H-^14^N OT-REDOR (grey) with *f* = 0.5 (complete saturation) and 1.0 (complete inversion) derived from Eqs 2,3, respectively, under the same ^1^H-^14^N dipolar coupling of 2.0 kHz. The optimum *τ*_mix_ of both curves are shown and highlighted by the dashed lines.

It is of practical use to clarify the differences between the universal expressions for ^1^H-^14^N RESPDOR and ^1^H-^14^N OT-REDOR. This can be done by comparing the universal curves resulting from Eqs. 2, 3 under the same *b*_1H–14N_. Figure 4 compares the three universal curves, one from Eq. (2) and two from Eq. (3) with *f* = 0.5 (complete saturation) and *f* = 1.0 (complete inversion). These two *f* values are chosen because they correspond to ideal RESPDOR (*f* = 0.5) and REDOR (*f* = 1.0) conditions and our working condition is an intermediate between these two, as mentioned in the *Introduction*. The two curves from Eq. (3) are identical except for the intensity (a factor of 2), which is in agreement with the discussion above (see Figure 4). A notable difference between the universal curves of ^1^H-^14^N OT and that of ^1^H-^14^N is that the dephasing rate of the former curves is about two times faster than that of the latter (0.77 ms compared to 1.60 ms, respectively). This is because, for ^1^H-^14^N OT, the REDOR effect is determined by the DQ transitions, whereas, for ^1^H-^14^N, the RESPDOR effect is determined by both the SQ and DQ transitions. Such a faster dephasing rate associated with the multiple quantum transitions has been known in the literature (Pruski et al., 1999). This potentially allows ^1^H-^14^N OT-REDOR to probe long ^1^H-^14^N distance better than ^1^H-^14^N RESPDOR as it is less affected by the poor sensitivity and uncertainty of Δ*S*/*S*_0_ at long *τ*_mix_.

## Results and Discussions

In this section, we firstly explore the feasibility of ^1^H-^14^N OT-REDOR on two model biological compounds of Tyr and AcAla. These two compounds only consist of a single ^14^N site and were well characterized by ^1^H-^14^N PM-S-RESPDOR in the previous study. Upon the feasibility test, we apply this technique to a more complex dipeptide system of AlaAla where two inequivalent ^14^N sites are present.

### L-Tyrosine.HCl

**FIGURE 5:**
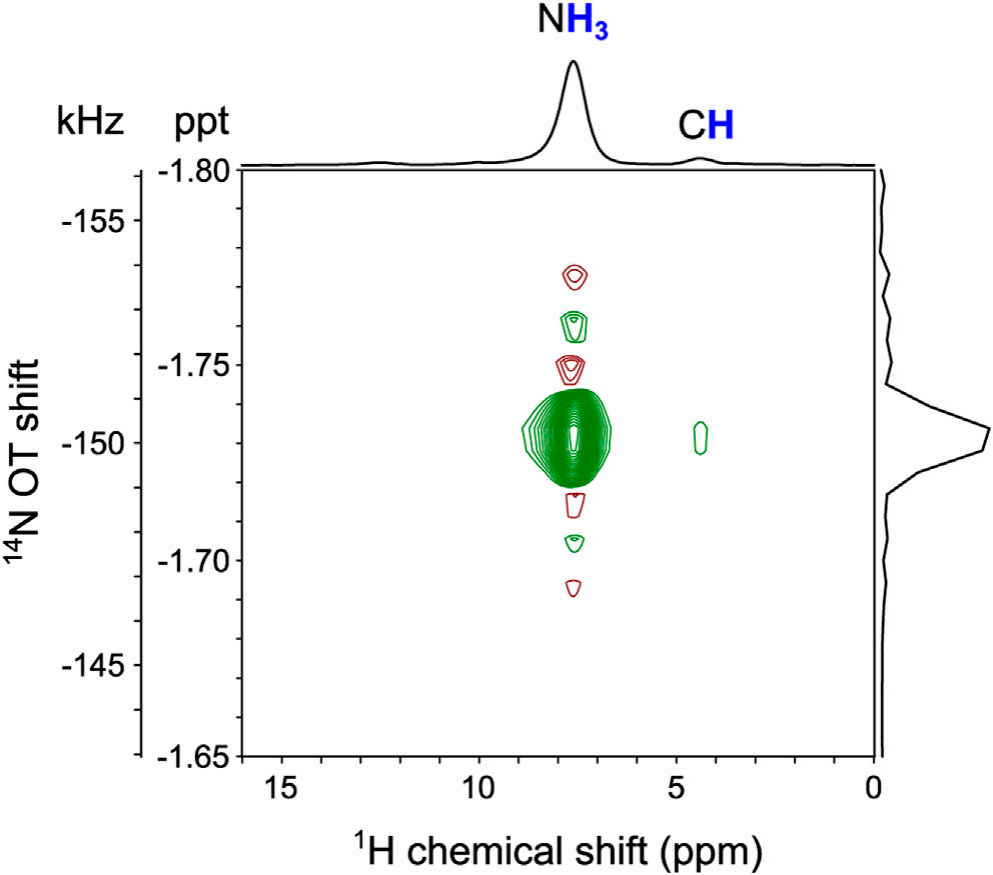
The 2D ^1^H-{^14^N OT} *D*-HMQC experiment of Tyr at the magnetic field (*B*_0_) of 14.1 T and *ν*_R_ of 62.5 kHz. The experiment was performed at the second ^14^N OT SSB (*n* = -2) for the highest S/N. Further details are given in the *Experiments* section.

In order to obtain an efficient ^1^H-^14^N OT-REDOR fraction curve, experimental optimizations are required. Such optimizations require the knowledge of ^14^N OT resonance frequency as it significantly affects the sensitivity of OT experiments due to the narrow bandwidth. In this current work, the ^14^N OT frequency is indirectly determined by the two-dimensional (2D) ^1^H-{^14^N OT} *D*- or T-HMQC (sequences in Supplementary Figures S1A,B respectively) at the second OT spinning sideband (*n* = -2 SSB) for the highest S/N. Such *n* value depends on the sense of rotation with respect to the magnetic field (Gan et al., 2018). For our configuration, the magnetic field is toward the top of the magnet and the spinning rotation is clockwise looking from the top. Taking benefits of optimum S/N, all the remaining experiments were also performed at the second ^14^N OT SSB (*n* = −2). Figure 5 clearly indicates the ^14^N OT frequency and its correlations to both proton sites of N**H**_**3**_ and C**H** by the *D*-HMQC experiment. The smaller correlation of N to C**H** under *τ*_mix_ of 0.51 ms is explained due to the longer ^1^H-^14^N distance compared to the directly bonded H-N distance of the NH_3_ group. This 2D spectrum is in agreement with the ^1^H-{^14^N} *D*-HMQC spectrum in the previous study (Duong et al., 2019).

**FIGURE 6:**
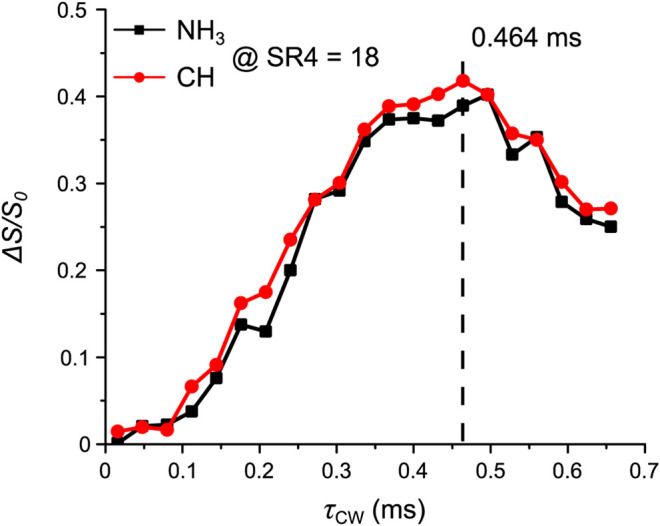
Tyr: the signal fraction *ΔS*/*S*_0_ of N**H**_**3**_ (black squares) and C**H** (red circles) as a function of *τ*_CW_. Experiments were performed at *τ*_mix_ of 1.15 ms. The optimum *τ*_CW_ is shown and highlighted by the dashed line. Further experimental details are given in the *Experiments* section.

After the ^14^N OT frequency has been determined, the next parameter for optimization is *τ*_CW_ so that as many as possible ^14^N crystallites can be saturated/inverted. The ^14^N OT rf-field was 120 kHz, the highest technically possible value. It was calibrated by the use of the Bloch–Siegert shift of the proton approach (Hung et al., 2020). However, it is noted that the ^14^N OT nutation frequency is scaled on *C*_Q_, the magnetic field, the powder distribution, making it much weaker than ^14^N OT rf-field. Figure 6 shows the signal fraction Δ*S*/*S*_0_ of N**H**_**3**_ and C**H** at a fixed *τ*_mix_ of 1.15 ms (or 18 loops of SR4^2^_1_ recoupling blocks) under varying *τ*_CW_ values.

**FIGURE 7:**
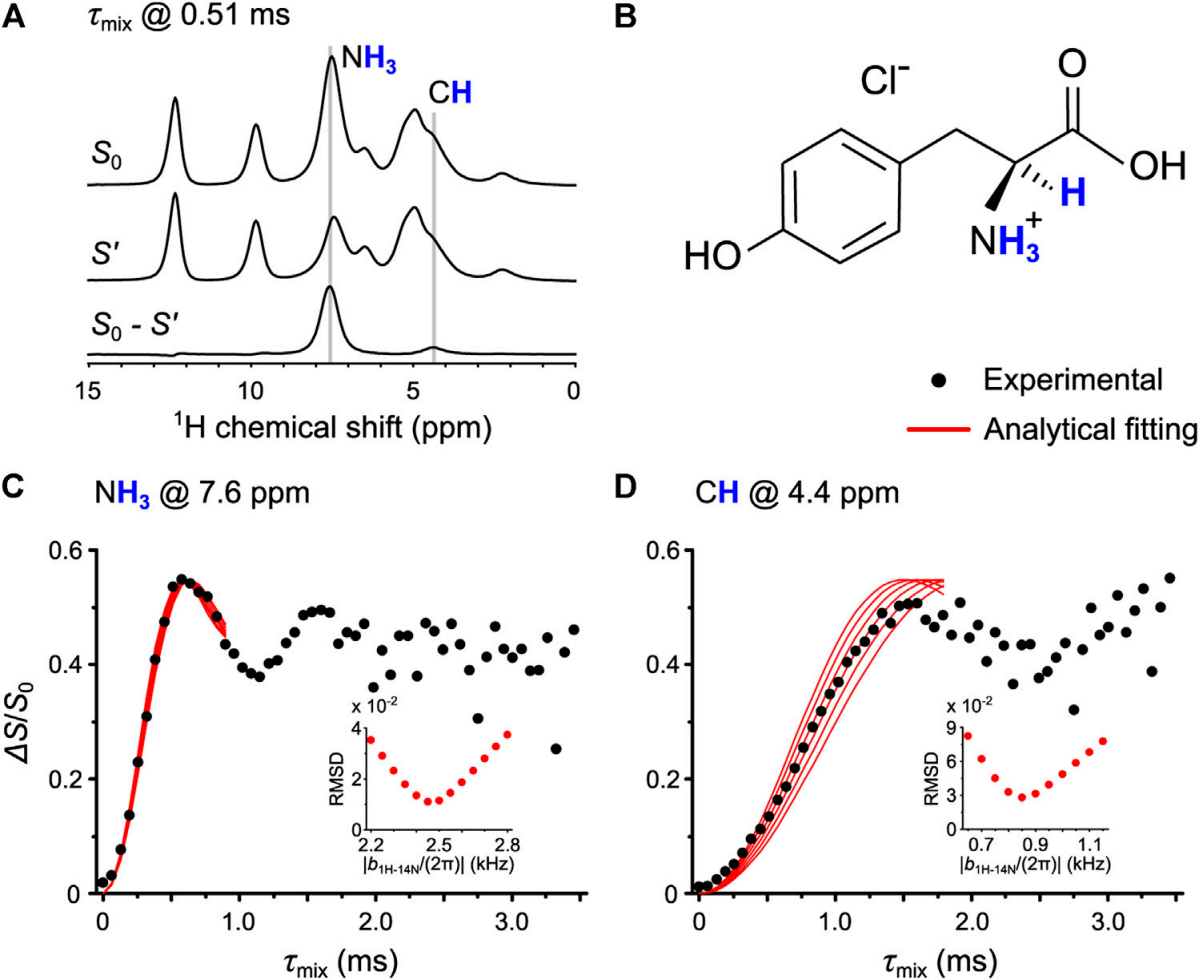
**(A)** The 1D ^1^H spin echo (*S*_0_), the dephased (*S*′), and the difference (*S*_0_–*S*′) spectra of Tyr. The former two were extracted from ^1^H-^14^N OT-REDOR with *τ*_mix_ of 0.51 ms, whereas the latter is calculated from the former two. **(B)** The molecular structure of Tyr where N**H**_**3**_ and C**H** are presented. (**C,D)** The fitting of experimental ^1^H-^14^N OT-REDOR fraction curves (black circles) by the universal curves (red lines) for N**H**_**3**_ at 7.6 ppm in **(C)** and C**H** at 4.4 ppm in **(D)**. The fitting parameter *f* for universal curves is 0.82. The RMSD analyses (inset) were calculated for the best fitting ^1^H-^14^N dipolar couplings.

Once the parameters were optimized, we performed the ^1^H-^14^N OT-REDOR experiment on Tyr. Figure 7A shows the one-dimensional (1D) ^1^H spin echo (*S*_0_), the dephased (*S*′), and the difference (*S*_0_–*S*’) spectra, in which the former two were extracted from the ^1^H-^14^N OT-REDOR experiment with *τ*_mix_ of 0.51 ms, whereas the latter is calculated from the former two. The N**H**_**3**_ and C**H** sites are evidently assigned in Figure 7A and are located in the molecular structure of Tyr (Figure 7B). The experimental fraction curves (black circles) of N**H**_**3**_ and C**H** are shown in Figures 7C,D, respectively. There are two important points to mention for the fraction curve of N**H**_**3**_ in Figure 7C. First, the oscillation is observed and the maximum Δ*S*/*S*_0_ is reached at *τ*_mix_ of 0.6 ms. In our previous study, the ^1^H-^14^N PM-S-RESPDOR fraction curve shows the maximum at *τ*_mix_ of ∼1.4 ms (Duong et al., 2019), which is about two times slower than that of ^1^H-^14^N OT-REDOR fraction curve. This result is in qualitative agreement with the analysis in *Pulse Sequence and the Universal Expression* and Figure 4. Second, the fraction curve in Figure 7C shows the experimental maximum Δ*S*/*S*_0_ of 0.55, smaller than the theoretical maximum of 0.67 by the universal curve in Eq. (3) for complete inversion. Thus, the fitting parameter *f* of 0.55/0.67∼0.82 is required for the reliable fittings. In addition, the value of 0.82 is close to *f* = 1 in the case of complete inversion, revealing the dominance of the inversion process under the application of ^14^N OT CW pulse. Once *f* is determined, the only unknown remaining parameter is *b*_1H–14N_. Moreover, according to the fitting strategy in *Pulse Sequence and the Universal Expression*, this fitting parameter *f* can also be used in Figure 7D. This is because *f* only depends on ^14^N *C*_Q_, *τ*_CW_, and ^14^N OT nutation frequency (which are the same as fraction curves in Figures 7C,D are from the single experiment) and thus should remain the same for other ^1^H-^14^N pairs from the same ^14^N site. The ^1^H-^14^N dipolar couplings, thus distances, can be extracted by fitting the scaled universal curves (red solid lines) to the experimental fraction curves (black circles) presented in Figures 7C,D. Although the fraction curve in Figure 7C shows the oscillation up to *τ*_mix_ of ∼1.8 ms, the fitting by the universal curves is only up to *τ*_mix_ of ∼1.0 ms, owing to the poor agreement between the experimental and universal curves for NH_3_ at *τ*_mix_ > 1.0 ms (Supplementary Figure S2A). The deviation is mainly caused by the fact that each crystallite experiences different ^14^N OT saturation/inversion extent depending on its relative orientation between quadrupolar tensor to the rotor-fixed frame, whereas, for the universal approach, the behaviors of the entire crystallites are considered uniform. The root-mean-square deviation (RMSD) in Figures 7C,D was calculated for the best fit of ^1^H-^14^N dipolar couplings. It is noted that, for N**H**_**3**_ (Figure 7C), a scaling factor P_2_(cos(θ)) (θ, the angle between H-N and C-N, is 109.5°) is used for the dynamic average of the N-H dipolar coupling due to the threefold rotation. The ^1^H-^14^N distances by OT-REDOR are shown in Table 1 along with those by PM-S-RESPDOR and neutron diffraction (ND). The distances are in good agreement with each other, which demonstrates the feasibility of OT-REDOR for obtaining accurate ^1^H-^14^N distances. It is worth noting that the longer distances by ssNMR than those from neutron result from the different vibrational averages of the internuclear distances of the two techniques (Ishii et al., 1997).

**TABLE 1:** ^1^H-^14^N distances measured by OT-REDOR, PM-S-RESPDOR, and ND on Tyr.

	OT-REDOR	PM-S-RESPDOR	ND
**NH**_**3**_	1.05 ± 0.03 Å	1.16 Å	1.01 Å
**CH**	2.21 ± 0.10 Å	2.24 Å	2.10 Å

### N-Acetyl-L-alanine

**FIGURE 8:**
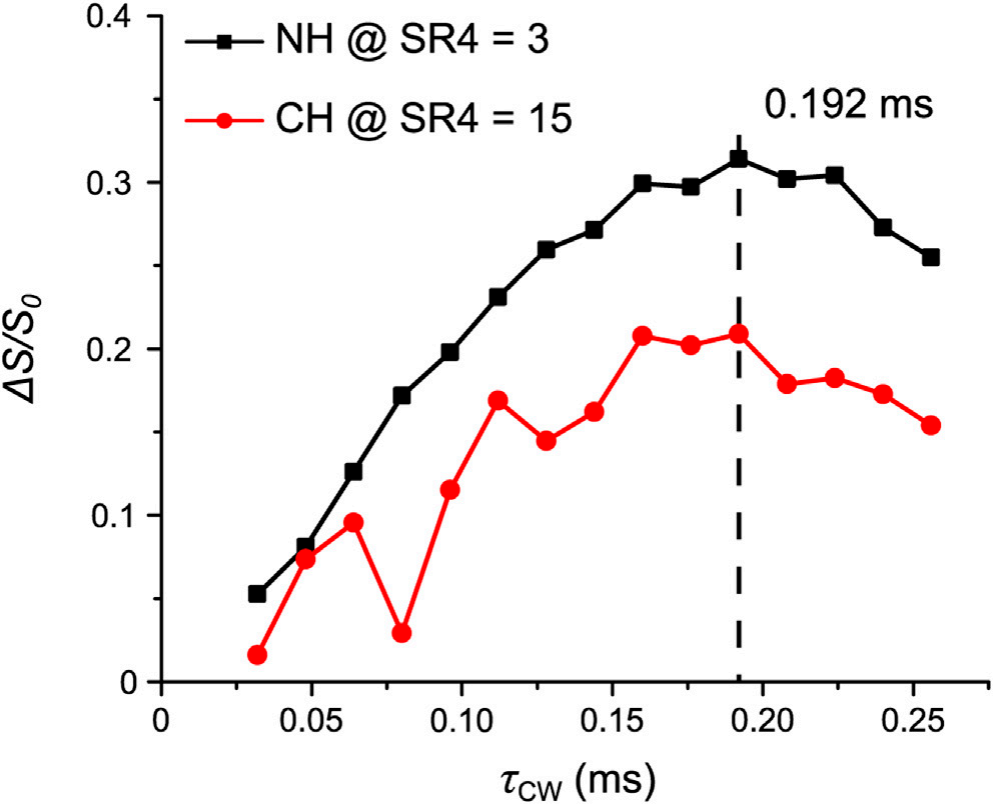
AcAla: the signal fraction *ΔS*/*S*_0_ of N**H** (black squares) at *τ*_mix_ of 0.19 ms and C**H** (red circles) at *τ*_mix_ of 0.96 ms as a function of *τ*_CW_. The optimum *τ*_CW_ is shown and highlighted by the dashed line. Further experimental details are given in the *Experiments* section.

To further demonstrate the feasibility of OT-REDOR for a system with a larger ^14^N *C*_Q_, we apply it to AcAla. A similar experimental procedure as described for Tyr was applied, including the 1) determination of ^14^N OT resonance frequency, 2) optimization of *τ*_CW_, and 3) implementation of OT-REDOR. These experiments were all performed at the second ^14^N OT SSB for the highest S/N. For step 1, the ^1^H-{^14^N OT} T-HMQC was performed (Supplementary Figure S3). Again, for the efficient OT-REDOR fraction curve, *τ*_CW_ must be optimized. For step 2, such optimization for N**H** (black squares) at *τ*_mix_ of 0.19 ms (or three loops of SR4^2^_1_ recoupling blocks) and C**H** (red circles) at *τ*_mix_ of 0.96 ms (or 15 loops of SR4^2^_1_ recoupling blocks) under identical *τ*_CW_ range is shown in Figure 8. The reason for different *τ*_mix_ is due to the large difference between ^1^H-^14^N distances for these proton sites. The optimum *τ*_CW_ of 0.192 ms for ^**14**^ **N**H in AcAla is shorter than *τ*_CW_ of 0.464 ms for ^**14**^ **N**H_3_ in Tyr. This result is expected since the ^14^N site of NH has a larger quadrupolar interaction, thus resulting in a larger ^14^N OT nutation field and shorter pulse length for efficient saturation/inversion.

**FIGURE 9:**
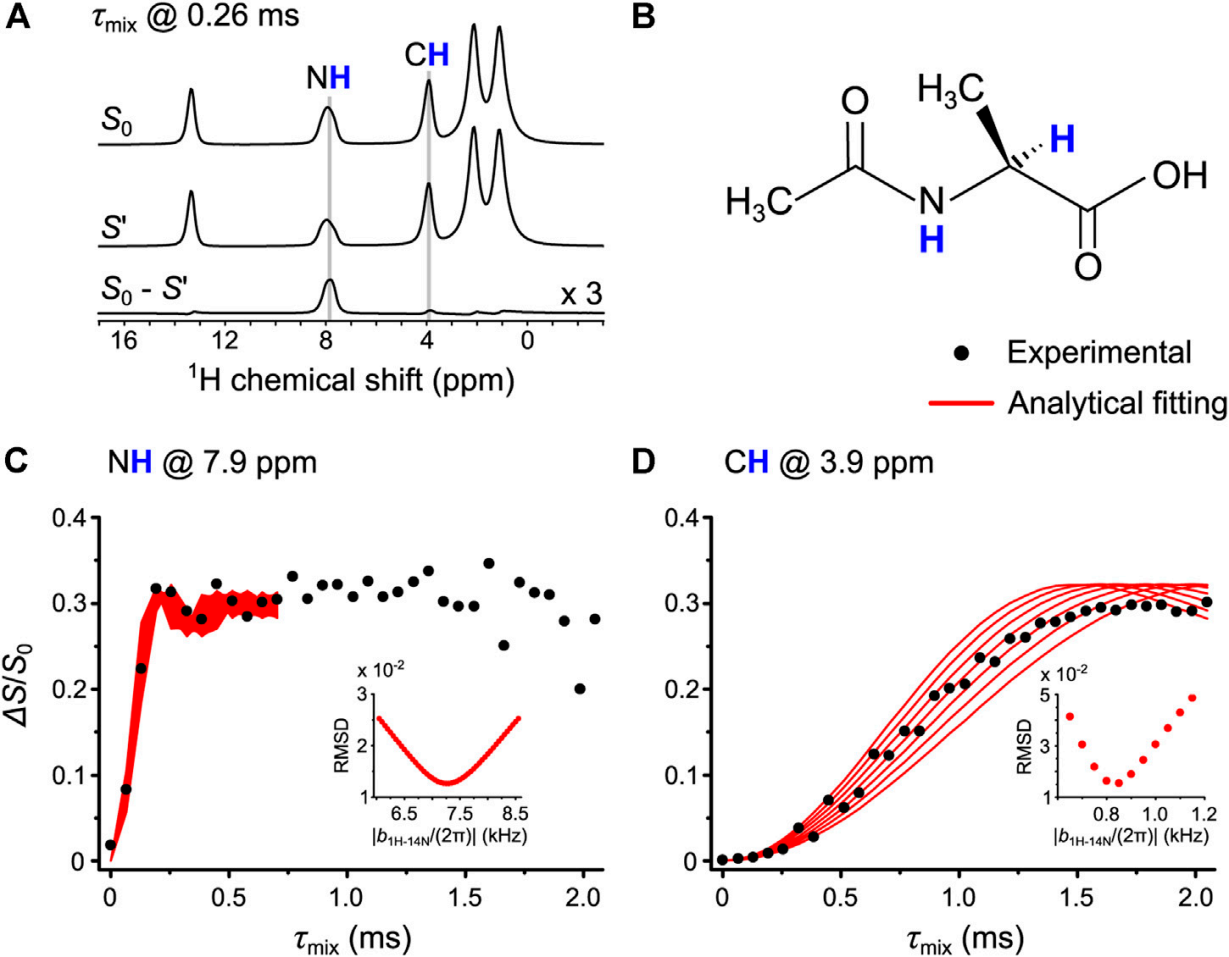
**(A)** The 1D ^1^H spin echo (*S*_0_), the dephased (*S*′), and the difference (*S*_0_–*S*′) spectra of AcAla. These spectra result from the ^1^H-^14^N OT-REDOR experiment with *τ*_mix_ of 0.26 ms. For readability, the intensity of the difference spectrum is triple. **(B)** The molecular structure of AcAla where N**H** and C**H** are located. (**C,D)** The fitting of experimental ^1^H-^14^N OT-REDOR fraction curves (black circles) by the universal curves (red lines) for N**H** at 7.9 ppm in **(C)** and C**H** at 3.9 ppm in **(D)**. The fitting parameter *f* for universal curves is 0.50. The RMSD analyses (inset) were calculated for the best fitting ^1^H-^14^N dipolar couplings.

For step 3, these parameters were used for the OT-REDOR experiment on AcAla. Figure 9A shows the one-dimensional (1D) ^1^H spin echo (*S*_0_), the dephased (*S*′), and the difference (*S*_0_–*S*′) spectra. The former two were extracted from the ^1^H-^14^N OT-REDOR experiment with *τ*_mix_ of 0.26 ms, whereas the latter results from the difference of the former two. For readability, the intensity of the difference spectrum is triple, showing the N**H** and C**H** sites which experience the REDOR effect. These two sites are also located in the molecular structure of AcAla (Figure 9B). Figures 9C,D show the fittings between the universal curves (red lines) and the experimental fraction curves (black circles) for N**H** and C**H**, respectively. For reliable fittings, the fitting parameter *f* must be known. From Figure 9C, the experimental maximum Δ*S*/*S*_0_ intensity of 0.33 results in *f* of 0.33/0.67 = 0.50. While Tyr shows the dominance of inversion (*f* = 0.82), AcAla experiences the saturation of overall magnetization (*f* = 0.50). The difference may arise from the large frequency linewidth (up to 8.6 kHz in Supplementary Figure S3) of ^14^N OT spectrum of NH of AcAla relative to the weak ^14^N OT nutation frequency. Since both fraction curves in Figures 9C,D were obtained from the single experiment where ^14^N *C*_Q_, *τ*_CW_, and ^14^N OT nutation frequency are the same, the identical *f* value can be used in Figure 9D. The extracted ^1^H-^14^N distances are summarized in Table 2 along with those from PM-S-RESPDOR and XRD. The distances are in agreement with each other. We note that the deviation between the distance of N–H by XRD and those by OT-REDOR and PM-S-RESPDOR is due to the poor capability of XRD to locate H position, resulting from the limited scattering power of hydrogen and the vibrational effect mentioned in *L-Tyrosine.HCl*. In short, the applicability of OT-REDOR on Tyr and AcAla for obtaining accurate ^1^H-^14^N distances has been validated.

**TABLE 2:** ^1^H-^14^N distances measured by OT-REDOR, PM-S-RESPDOR, and XRD on AcAla.

	OT-REDOR	PM-S-RESPDOR	XRD
**NH**	1.07 ± 0.06 Å	1.06 Å	0.78 Å
**CH**	2.21 ± 0.12 Å	2.11 Å	2.09 Å

### L-Alanyl-L-alanine

**FIGURE 10:**
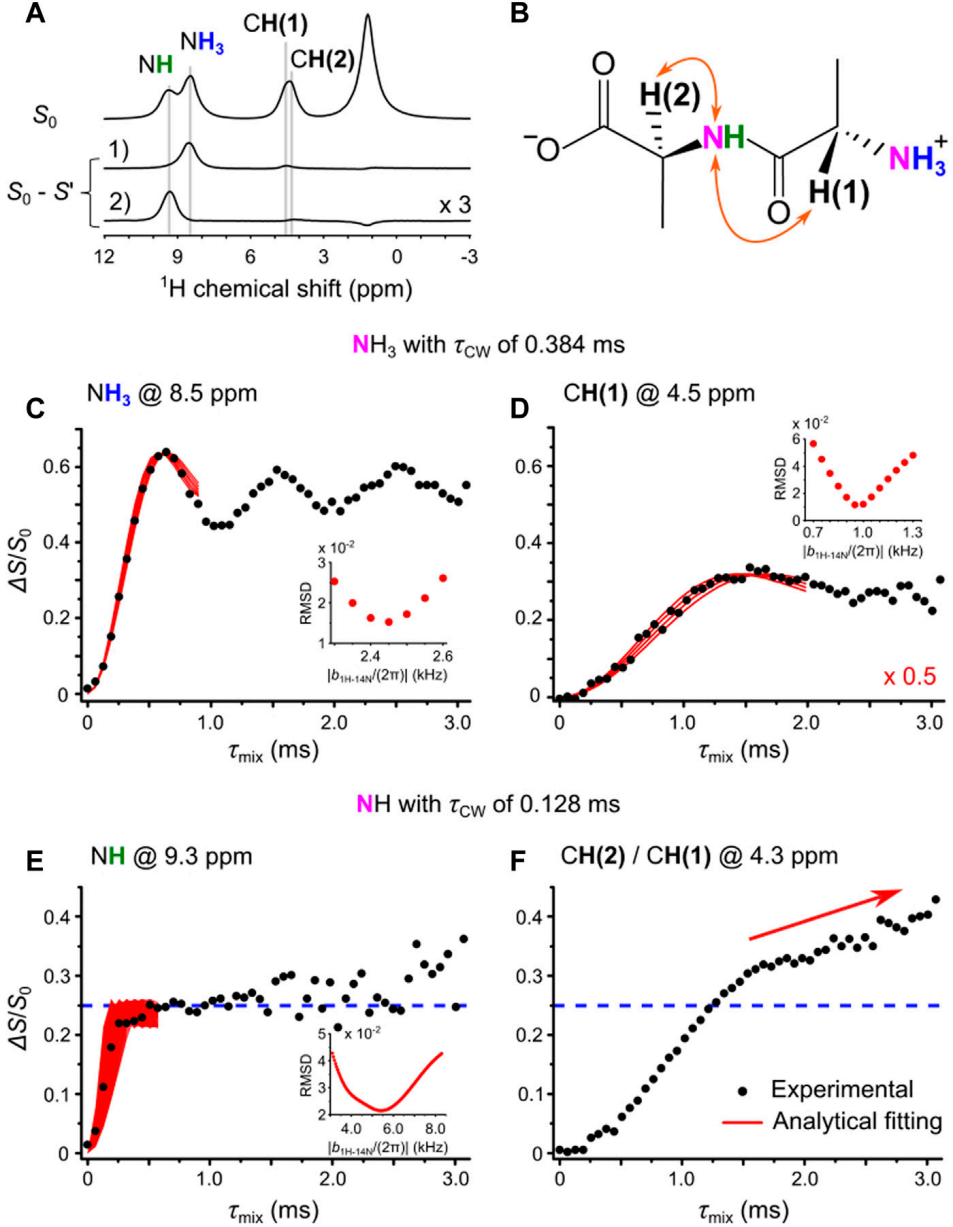
**(A)** The 1D ^1^H spin echo and the two difference spectra where 1) N**H**_**3**_ and 2) N**H** sites are saturated/inverted during ^1^H-^14^N OT-REDOR experiments with *τ*_mix_ of 0.51 ms and 0.19 ms, respectively. For readability, the intensity of spectrum 2) is triple. **(B)** The N**H**, N**H**_**3**_, C**H(1)**, and C**H(2)** sites, assigned in **(A)**, are located in the molecular structure of AlaAla. **(C–F)** The fitting of experimental ^1^H-^14^N OT-REDOR fraction curves (black circles) by the universal curves (red lines) for N**H**_**3**_ at 8.5 ppm in **(C)** and C**H(1)** at 4.5 ppm in **(D)** when ^**14**^ **N**H_3_ OT was saturated/inverted by CW pulse with *τ*_CW_ of 0.384 ms and N**H** at 9.3 ppm in **(E)** when ^**14**^ **N**H OT was saturated/inverted by CW pulse with *τ*_CW_ of 0.128 ms. The fitting parameter *f* for universal curves is (**C,D)** 0.94 and (**E)** 0.37. For **(D)**, the universal curves are halved, highlighted by a factor of 0.5 in red. For **(E)**, the maximum signal fraction *ΔS*/*S*_0_ of 0.25 for N**H** is presented by the horizontal dash line. For **(F)**, the signal fraction *ΔS*/*S*_0_ of C**H(2)**/C**H(1)** at 4.3 ppm with ^**14**^ **N**H OT irradiation is over this line and is still increasing with *τ*_mix_ (highlighted by the arrow). The RMSD analyses (inset) were calculated for the best fitting ^1^H-^14^N dipolar couplings. The two arrows show the proximity of both C**H(2)**/C**H(1)** to ^**14**^ **N**H site.

In the previous section, OT-REDOR experiments have been successfully applied to Tyr and AcAla, both containing a single ^14^N site. As the main usage of OT-REDOR is for systems where multiple ^14^N sites are present, here, we apply this sequence to AlaAla. Besides the 1D ^1^H spin echo (*S*_0_) at the top, Figure 10A also shows the two difference (*S*_0_–*S*’) spectra where 1) N**H**_**3**_ and 2) N**H** sites are saturated/inverted during ^1^H-^14^N OT-REDOR experiments with *τ*_mix_ of 0.51 ms and 0.19 ms, respectively. The N**H**, N**H**_**3**_, C**H(1)**, and C**H(2)** sites are unambiguously assigned (Figure 10A) and located in the molecular structure of AlaAla (Figure 10B). This compound consists of two ^14^N sites of **N**H_3_ and **N**H; hence, it is similar to the combination of ^14^N sites of Tyr and AcAla.

Similar experimental procedures were applied. *D*-HMQC experiments were performed again at the second ^14^N OT SSB (*n* = -2) for the highest S/N to determine ^14^N OT frequencies (Supplementary Figures S4A,B **)**. Then, *τ*_CW_ for ^**14**^ **N**H_3_ and ^**14**^ **N**H of AlaAla were optimized, which were 0.384 and 0.128 ms, respectively (Supplementary Figures S4C,D). These optimized values, similar to those of Tyr and AcAla, were used to obtain ^1^H-^14^N OT-REDOR fraction curves.

We discuss first the case where ^**14**^ **N**H_3_ is saturated/inverted by CW pulse with *τ*_CW_ of 0.384 ms. The experimental fraction curves of N**H**_**3**_ and C**H(1)** (black circles) are shown in Figures 10C,D, respectively. For N**H**_**3**_, the experimental curve shows the oscillation and the maximum Δ*S*/*S*_0_ is 0.63 (see Figure 10C). Thus, the fitting parameter *f* of 0.63/0.67∼0.94 for universal curves is required for reliable fittings. This indicates that an almost complete inversion of ^14^N OT is achieved (*f* = 1), similar to the case of NH_3_ of Tyr. Another similarity to Tyr is that significant deviation is observed, especially at long *τ*_mix_ for the full-scale fitting between the fraction curve of Figure 10C and the universal curves (see Supplementary Figure S2B). For C**H(1)**, the fraction curve reaches the plateau *ΔS*/*S*_0_ of 0.35 at *τ*_mix_ of 1.50 ms (Supplementary Figure S5). The reason for the lower signal fraction *ΔS*/*S*_0_ is the overlapping of ^1^H signals of C**H(1)** and C**H(2)** sites, in which only C**H(1)** is close to ^**14**^ **N**H_3_. For a good match with the experimental fraction curve, we need to halve the universal curves in Figure 10D. The reason for using a factor of 0.5 is explained in the Supplementary Eq. S3. Without scaling, the obtained ^1^H-^14^N distance by NMR is in poor agreement with that reported from XRD (see Supplementary Figure S5). The fitting of the scaled universal curves to the ^1^H-^14^N OT-REDOR fraction curves gives the ^1^H-^14^N distances of 1.05 ± 0.03 Å for NH_3_ (after a modulation factor of P_2_(cos(θ)) as described in *L-Tyrosine.HCl* and *Pulse Sequence and the Universal Expression* and 2.09 ± 0.05 Å for CH(1) (after halving the universal curves). Both measured distances are in good agreement with those reported from XRD (see Table 3).

**TABLE 3:** ^1^H-^14^N distances measured by OT-REDOR and XRD on AlaAla.

	Saturation/inversion on ^14^N(H_3_)		Saturation/inversion on ^14^N(H)
	NH_3_	CH(1)	NH
**OT-REDOR**	1.05 ± 0.03 Å	2.09 ± 0.05 Å	1.19 ± 0.20 Å
**XRD**	0.93 Å	1.97 Å	0.96 Å

We then consider the case where ^**14**^ **N**H is saturated/inverted by CW pulse with *τ*_CW_ of 0.128 ms. The fraction curves of N**H** and C**H(2)**/C**H(1)** (black circles) are shown in Figures 10E,F, respectively. For N**H** in Figure 10E, the experimental curve shows the plateau Δ*S*/*S*_0_ of 0.25 at *τ*_mix_ of 0.50 ms and then large fluctuations of Δ*S*/*S*_0_ at *τ*_mix_ larger than 1.50 ms. The origin of such fluctuations may be due to *t*_1_ noise from spinning frequency fluctuation (Nishiyama et al., 2020). As the maximum Δ*S*/*S*_0_ is smaller than the theoretical maximum of 0.67 of the universal curve, a fitting parameter *f* of 0.25/0.67∼0.37 is required. Based on the fittings of the universal curves (red solid lines) to the ^1^H-^14^N OT-REDOR fraction curve, the ^1^H-^14^N distance is measured to be 1.19 ± 0.20 Å. This value is in excellent agreement with ^1^H-^15^N distance by inverse cross-polarization with variable contact (CPVC) (Nishiyama et al., 2016) (Supplementary Figure S6) and in close agreement with the distance of 0.96 Å by XRD (see Table 3). An advantage of ^1^H-^14^N OT-REDOR compared to ^1^H-^15^N inverse CPVC is that multiple H-N distances can be simultaneously determined by the former, whereas only directly bonded distance is determined by the latter due to the dipolar truncation effect. Indeed, ^1^H-^15^N inverse CPVC only provides the distance of directly bonded H-N for the NH site (see Supplementary Figure S6). For C**H(2)**/C**H(1)** fraction curve, its fraction signal Δ*S*/*S*_0_ is larger than 0.25 and continues to grow at long *τ*_mix_ (see Figure 10F). Although this curve is the combination of two curves because there are two C**H** groups that are close to ^**14**^ **N**H site and their ^1^H chemical shifts are overlapped, Δ*S*/*S*_0_ is larger than 0.25 may result from the intermolecular couplings. Because of this complexity, we did not fit this with the universal curves.

In conclusion, for AlaAla, the ^1^H-^14^N OT-REDOR experiment can be used for the accurate measurement of ^1^H-^14^N distances for the bonded H–N distances of each nitrogen. However, extracting distances for nonbonded H-N pairs is still difficult, especially when the chemical shifts of these ^1^H sites are overlapped, as shown in Figure 10F. Such problem will be solved by the multidimensional NMR experiments, for example, with an addition of the ^13^C dimension.

## Conclusion

In summary, we have presented the feasibility of ^1^H-^14^N OT-REDOR with proton detection at fast MAS to extract ^1^H-^14^N distances for Tyr, AcAla, and AlaAla. Owing to the selective characteristics of ^14^N OT spectroscopy, this sequence is useful for systems with multiple ^14^N sites. Other advantages of ^14^N OT are the availability of commercial MAS probes, the robustness of misadjustment of the magic angle, and the fast dephasing rate. The final advantage is that it allows probing longer ^1^H-^14^N distances better than ^1^H-^14^N RESPDOR experiment. For efficient ^1^H-^14^N OT-REDOR fraction curve, the ^14^N OT resonance frequency, in this work, must be determined with *D*- or T-HMQC experiments and the CW pulse length must be optimized. For reliable ^1^H-^14^N distances, the fitting parameter *f* is a prerequisite; otherwise, distances cannot be accurately determined. The knowledge of *f* value also enables the evaluation of saturation/inversion degree of ^14^N OT by the CW pulse. For Tyr and AcAla compounds, the extracted distances from OT-REDOR are in good agreement with PM-S-RESPDOR and the diffraction techniques. For AlaAla, the extracted ^1^H-^14^N distances from directly bonded N–H well agree with those reported from XRD and ^1^H-^15^N inverse CPVC. However, this is not the case for nonbonded N–H pairs since distance deviations from those reported by XRD are observed. The reason for such deviation is the overlapping of ^1^H signals. This issue can be overcome by performing multidimensional NMR experiments. In conclusion, we believe that the ^1^H-^14^N OT-REDOR has the potential of selectively measuring ^1^H-^14^N distances on systems containing multiple ^14^N sites, giving deep insights into structural studies of biological, chemical, and pharmaceutical compounds. It is worth noting that ^14^N selective saturation can also be achieved in the manner of DANTE. It is promising to perform ^1^H-^14^N DANTE-RESPDOR experiments in future studies.

## Experiments

L-tyrosine.HCl (Tyr), N-acetyl-L-alanine (AcAla), and L-alanyl-L-alanine were purchased from Sigma-Aldrich and used as received. The samples were separately packed into 1.0 mm zirconia rotors and then inserted into 1 mm ^1^H/X double-resonance probe. The rotors were spun at a MAS frequency of 62.5 kHz, except for ^1^H-^15^N inverse CPVC at 70 kHz.

All NMR experiments were recorded at a room temperature of 25 °C on JNM-ECZ600R (JEOL RESONANCE Inc.) at 14.1 T solid-state NMR spectrometers. The ^1^H and ^14^N OT Larmor frequencies are 600.0 and 86.8 MHz, respectively. For the highest S/N, the ^14^N OT frequency was set at the second SSB (*n* = −2). The ^14^N and ^14^N OT shifts are referenced to CH_3_NO_2_, whose ^14^N and ^14^N OT shifts are equal to 0 ppm or 0 kHz. The ^1^H rf-field was 328 kHz for π/2 and π pulses and 140 kHz for the SR4^2^_1_ recoupling sequence. The ^14^N OT rf-field was 120 kHz.

For Tyr, the 2D ^1^H-{^14^N} *D*-HMQC spectrum in Figure 5 was recorded using the sequence shown in Supplementary Figure S1A with 8 scans, 32 *t*_1_ points, and rotor-synchronized *t*_1_ increment of 16.0 µs. *τ*_p_, *τ*_mix_, and recycling delay (RD) were 200 µs, 512 µs, and 4 s, respectively. The experimental time was about 0.6 h. The States-TPPI method was employed for quadrature detection along the indirect dimension. For Figure 6, *τ*_CW_ was optimized within the range from 16 µs to 656 µs with a step of 32 µs; the ^14^N OT frequency was −1.73 ppt (parts per thousand), the τ_mix_ was fixed at 1152 µs, the number of scans (NS) was 18, and RD was 5.0 s. The experimental time was 1.1 h. For Figure 7, the ^1^H-^14^N OT-REDOR was performed at *τ*_CW_ of 464 µs, ^14^N OT frequency of −1.73 ppt, NS of 72, RD of 6.5 s, and *τ*_mix_ from 0 to 3456 µs with a step of 64 µs. The experimental time was 14.3 h.

For AcAla, the *τ*_CW_ optimization in Figure 8 was implemented within the range from 32 µs to 256 µs with a step of 16 µs; the ^14^N OT frequency was −1.267 ppt, NS was 18 and RD was 10.0 s, and *τ*_mix_ was fixed at 192 µs for N**H** at 7.9 ppm and 960 µs for C**H** at 3.9 ppm. The experimental times for both experiments were 1.5 h. For Figure 9, the ^1^H-^14^N OT-REDOR was performed at *τ*_CW_ of 192 µs, ^14^N OT frequency of −1.267 ppt, NS of 108, RD of 10 s, and *τ*_mix_ from 0 to 2048 µs with a step of 64 µs. The experimental time was 19.8 h.

For AlaAla, the ^1^H-^14^N OT-REDOR experiments in Figure 10 were performed at NS of 144, RD of 2.5 s, *τ*_mix_ from 0 to 3072 µs with a step of 64 µs, and *τ*_CW_, ^14^N OT frequencies were of 384 µs, −1.72 ppt and 128 µs, −1.26 ppt for ^**14**^ **N**H_3_ and ^**14**^ **N**H, respectively. The experimental times for both experiments were 9.8 h.

NMR data are available upon request.

## Data Availability

The original contributions presented in the study are included in the article/Supplementary Material; further inquiries can be directed to the corresponding author.
