# Supplementary material for: Selective 1H-14N Distance Measurements by 14N Overtone Solid-State NMR Spectroscopy at Fast MAS
Source: Front Mol Biosci. 2021 Apr 8;8:645347. doi: 10.3389/fmolb.2021.645347 (PMC8061749; doi:10.3389/fmolb.2021.645347)
Supplement: Supplementary file 1 [file datasheet1.pdf]

SUPPORTING INFORMATION

Selective ^1H-^{14}N distance measurements by ^{14}N overtone solid-state NMR spectroscopy at fast MAS

Nghia Tuan Duong[1], Zhehong Gan[2], Yusuke Nishiyama[1,3]*

[1] NMR Science and Development Division, RIKEN SPring-8 Center, and Nano-Crystallography Unit, RIKEN-JEOL Collaboration Center, Yokohama, Kanagawa 230-0045, Japan

[2] Centre of Interdisciplinary Magnetic Resonance, National High Magnetic Field Laboratory, Tallahassee, Florida 32310, USA

[3] JEOL RESONANCE Inc., Musashino, Akishima, Tokyo 196-8558, Japan

* Corresponding author. Email: yunishiy@jeol.co.jp

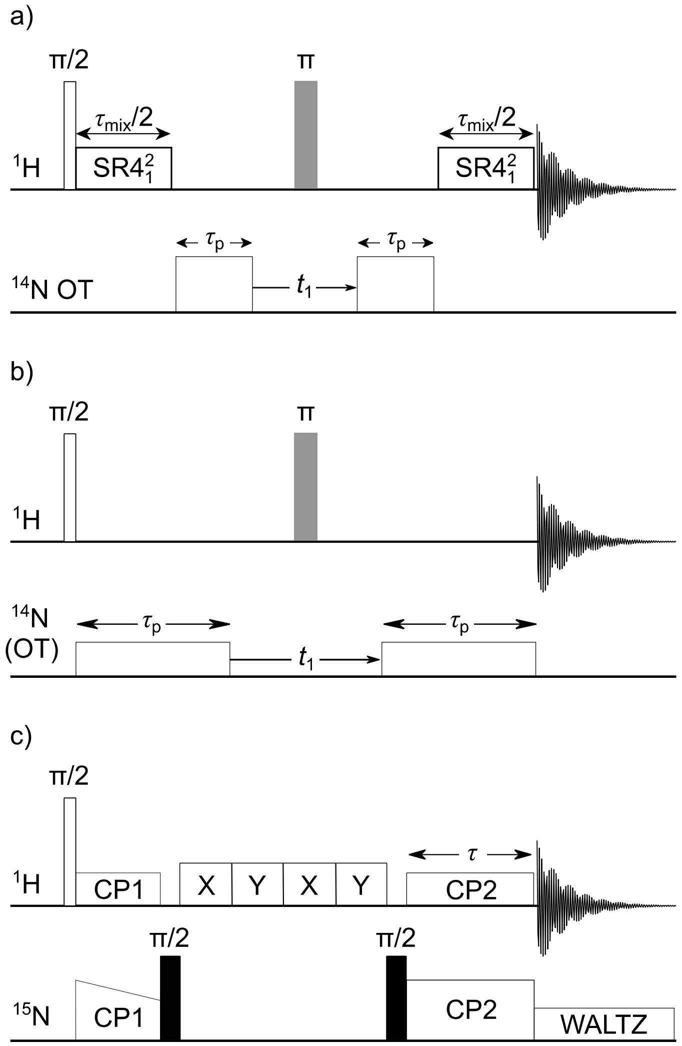

Figure S1. a) *D*-HMQC and b) T-HMQC sequences for probing ^{1}H-^{14}N OT correlations and c) ^{1}H-^{15}N inverse CPVC for determining ^{1}H-^{15}N dipolar couplings. All sequences are employed with ^{1}H-detection.

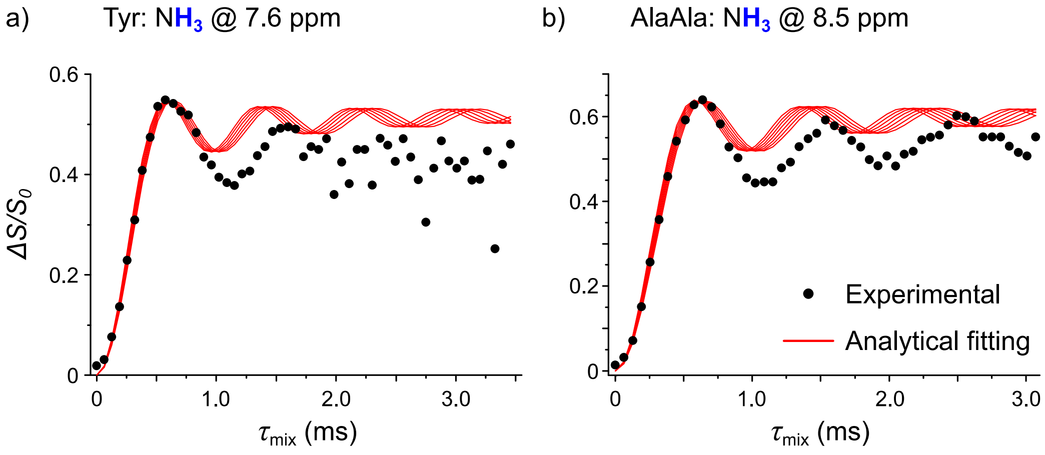

Figure S2. The full-scale fitting of experimental ^{1}H-^{14}N OT-REDOR fraction curves (black circles) by the universal curves (red lines) for a) Tyr: N**H₃** at 7.6 ppm and b) AlaAla: N**H₃** at 8.5 ppm. The fitting parameters *f* for universal curves are a) 0.82 and b) 0.94.

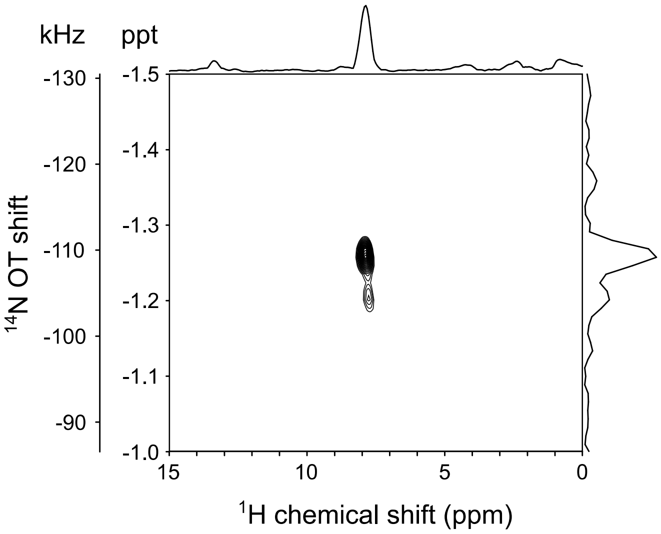

Figure S3. AcAla: the 2D ^{1}H-{^{14}N OT} T-HMQC spectrum at B_0 of 14.1 T and ν_R of 62.5 kHz. Experiment was recorded using the sequence in Fig. S1b with 8 scans, 16 t_1 points, and rotor-synchronized t_1 increment of 16.0 µs. The ^{14}N OT frequency was around the second SSB (n = -2) for the highest S/N. The τ_p and RD were 400 µs and 6 s, respectively. The experimental time was about 0.4 hour. The States-TPPI method was employed for the quadrature detection along the indirect dimension.

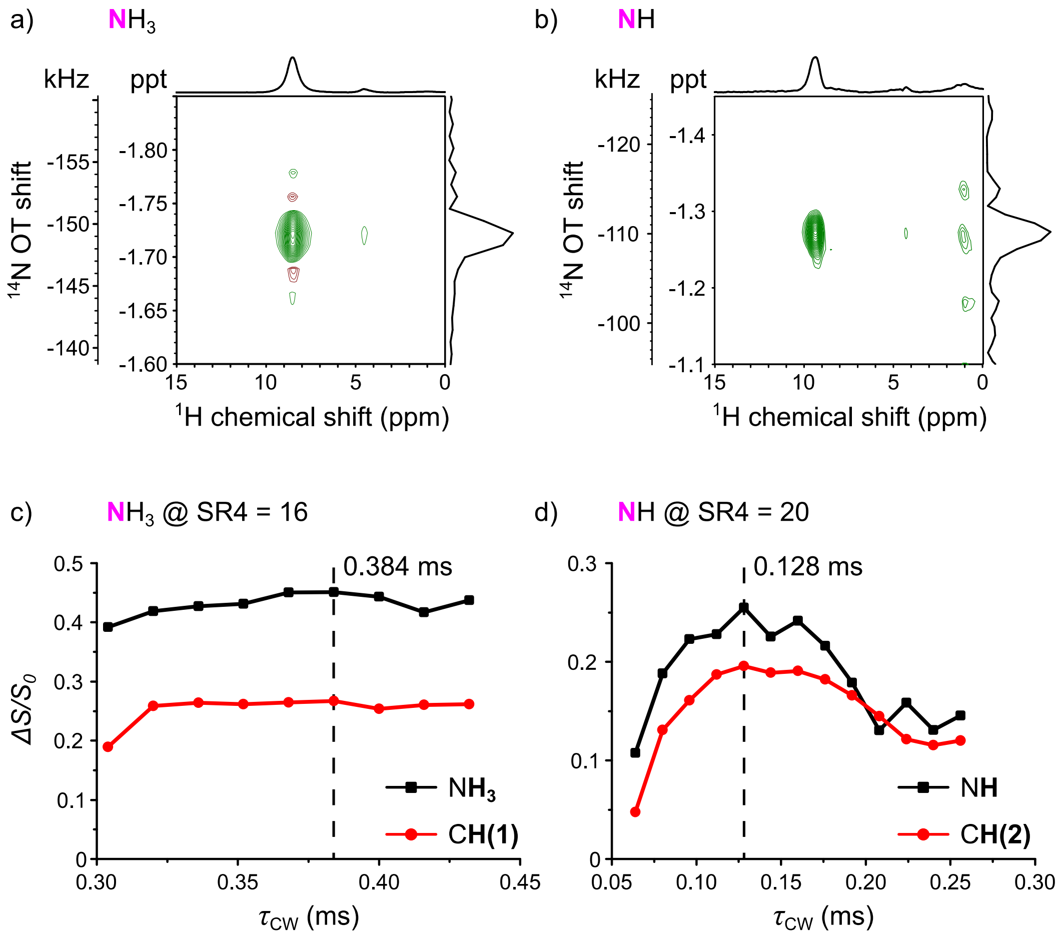

Figure S4. AlaAla: all experiments were performed at B_0 of 14.1 T and ν_R of 62. 5 kHz. (a,b) The 2D ^{1}H-{^{14}N OT} *D*-HMQC spectra for ^{14}**N**H_3 and ^{14}**N**H, respectively. Experiments were recorded using the sequence in Fig. S1a with 8 scans, 16 t_1 points, and rotor-synchronized t_1 increment of 16.0 µs. The ^{14}N OT frequencies were around the second SSB (n = -2) for the highest S/N. The (τ_p, τ_{mix}, RD) were (250 µs, 384 µs and 2 s) for a and (100 µs, 384 µs, and 2 s) for b. The States-TPPI method was employed for the quadrature detection along the indirect dimension. The experimental times for both 2D spectra were 0.15 hour. (c,d) The signal fraction $\Delta S/S_0$ as a function of τ_{CW} for c) N**H_3** (black squares) and C**H(1)** (red circles) at τ_{mix} of 1.02 ms when ^{14}**N**H_3 was saturated/inverted by τ_{CW} from 304 µs to 432 µs with a step of 16 µs at ^{14}N OT frequency of -1.72 ppt, and d) N**H** (black squares) and C**H(2)** (red circles) at τ_{mix} of 1.28 ms when ^{14}**N**H was saturated/inverted by τ_{CW} from 64 µs to 256 µs with a step of 16 µs at ^{14}N OT frequency of -1.26 ppt. NS and RD were 18 and 2s, respectively. The experimental times for were 0.2 and 0.3 hour for c and d, respectively. The optimum τ_{CW} is shown and highlighted by the dashed line.

Here we consider a spin system containing two protons and one nitrogen (H1 – N···H2), in which H1 is close to while H2 is far away from N. We assume the chemical shifts of H1 and H2 are overlapped to each other. Applying ^{1}H-^{14}N OT-REDOR to this three-spin system, we have:

Without CW pulse, the spin-echo signal S_0 is given by:

$$S_0 = S_0^{H1} + S_0^{H2} = 2S_0^{H1} \tag{S1}$$

As H2 is far away from N, its signal is not affected by the CW pulse. Hence, with the CW pulse, the dephased spin-echo signal S' is given by:

$$S' = S'^{H1} + S_0^{H2} = S'^{H1} + S_0^{H1} \tag{S2}$$

Combining Eqs. S1 and S2, the fraction signal is:

$$\frac{\Delta S}{S_0} = \frac{S_0 - S'}{S_0} = \frac{2S_0^{H1} - S'^{H1} - S_0^{H1}}{2S_0^{H1}} = \frac{S_0^{H1} - S'^{H1}}{2S_0^{H1}} = \frac{\Delta S^{H1}}{2S_0^{H1}} = \left(\frac{\Delta S}{S_0}\right)^{H1} / 2.0 \tag{S3}$$

Eq. S3 shows that due to the overlapping of H1 and H2, the actual H1-N fraction curve is scaled down by a factor of 2.0. Therefore, for a good match with the experimental curve, the universal curves should be also be halved.

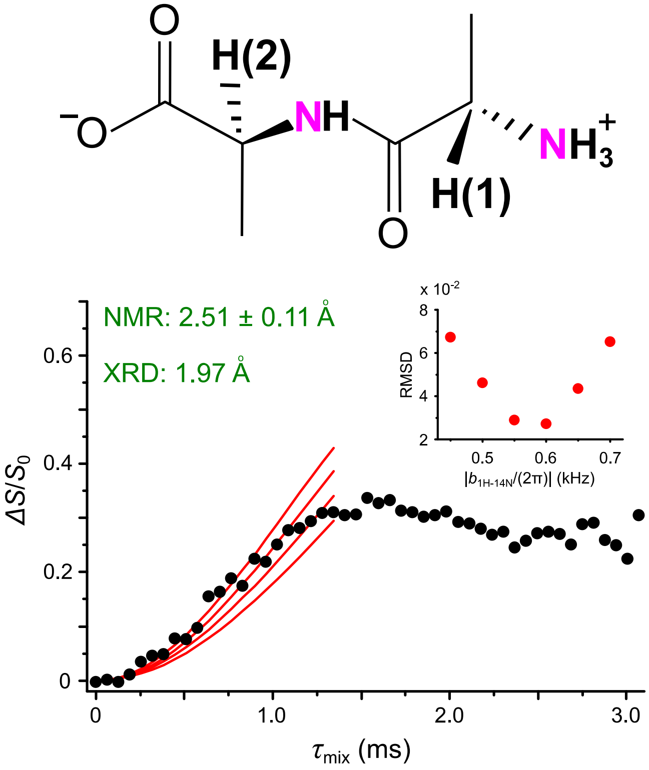

Figure S5. AlaAla (top): the fitting of experimental C**H(1)** fraction curves (black circles) by the universal curves (red lines) when $^{\mathbf{14}}\mathbf{N}H_3$ is saturated/inverted with τ_{CW} of 0.384 ms. The universal curve are not halved. The RMSD analyses (inset) were calculated for the best fitting ^{1}H-^{14}N dipolar couplings. The NMR and XRD distances are given. Experimental details are identical to those of Fig. 10d.

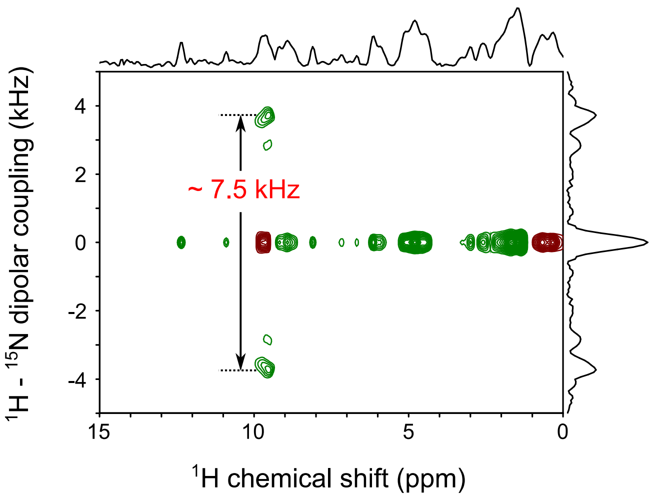

Figure S6. AlaAla: the 2D ^{1}H-^{15}N inverse CPVC spectrum. Experiment, using the sequence in Fig. S1c, was performed at B_0 of 14.1 T and ν_R of 70.0 kHz. The ^{15}N rf-field was 125 kHz. The ^{1}H → ^{15}N CP1 and ^{15}N → ^{1}H CP2 conditions were performed using ^{1}H and ^{15}N rf-fields of 20 and 50 kHz, respectively. For CP1, the linear ramp on ^{15}N channel was -10 % while for CP2, no linear ramp was used. The contact time of CP1 was 2.0 ms while that of CP2, τ, was varied from 0 to 1800 µs with a step of 10 µs. The 100 ms HORROR scheme with ν_{1H} = 35 kHz was used to suppress the residual ^{1}H polarizations after CP1. WALTZ decoupling, with ^{15}N rf-field of 10 kHz, was used to decouple nitrogen during ^{1}H acquisition. The ^{15}N chemical shift and its dimension were fixed at 80 ppm and 300 ppm, respectively. NS = 136 and RD = 2s. The experimental time was 13.7 hours. The ^{1}H-^{15}N dipolar coupling strength for NH site is determined by an arrow.

NMR pusle program:

```
--------------------------------------------------------------------------------
--	Experiment Source Code				--
--	Delta NMR Experiment & Machine Control Interface		--
--	Copyright (c) 2009 JEOL Ltd				--
--	All Rights Reserved				--
--------------------------------------------------------------------------------
--	HELP.eng: Hahn echo with optional decoupling
--	Category: solids, echo
--	File name : hahn_echo.ex2
--	Sequence name : Hahn echo with optional decoupling
--	Reference :
--	END HELP
header
	filename	=>	"respdor";
	sample_id	=>	"";
	comment	=>	"Hahn echo w/ opt decoupling";
	process	=	"1d_solid.list";
	include "header_solid";
end header;
instrument
	include "instrument_solid";
end instrument;
acquisition
	x_domain	=>	"Carbon13";
	x_offset	=>	100[ppm];
	x_sweep	=>	400[ppm];
	x_points	=>	2048;
	scans	=>	4;
	x_prescans	=>	0;
	mod_return	=>	1;
	include "acquisition_solid";
```

```
end acquisition;
pulse
	collect COMPLEX,OBS;
	include "pulse_solid";
	initial_wait	=	10.0[ms];
	irr_domain	=>	"Nitrogen14";
	irr_offset	=>	-300[ppm];

	obs_Setup	=?	"#Setup Observe Pulses#";
	obs_width_first =>	x90;
	obs_width_second=>	obs_width_first*2, help "second pulse width";
	obs_amp_pulse =>	100[%];
	obs_amp_sr4	=>	100[%];

	irr_Setup	=?	"#Setup Observe Pulses#";
	irr_width_sat	=>	x90,		help "first pulse width";
	irr_amp_pulse	=>	100[%], 0[%]->100[%]:0.01[%],	help "amplitude of pulses";
	irr_shape_sat	=>	"SQUARE",("SQUARE","PM_sat");

	Echo	=? "#Setup up echo times#";
	spinning_freq	=>	10[kHz];
	cycle_time_MAS=	1/spinning_freq;
	number_r4	=>	10;
	number_saturation=?	upper(irr_width_sat*spinning_freq);
	include "obs_sat_solid";

	recycle_Setup	=? "#Setup Recycle Times#";
	relaxation_delay=>	5.0[s],		help "relaxation delay";
	repetition_time =?	relaxation_delay + x_acq_time,  help"relaxation_delay+x_acq_time";

	atn_Setup	=? "#Experiment Attenuator Settings#";
	obs_atn		=>	xatn,		help "attenuator for obs";
```

```
irr_atn          =>    irratn,          help "attenuator for irr";

obs_phs_first    =     {3(0), 3(120), 3(240)};
obs_phs_sr4      =     {0};
obs_phs_second=        {0, 120, 240};
irr_phs_sat      =     {0};
obs_phs_acq      =     {0, 240, 120, 240, 120, 0, 120, 0, 240};
module_config  = "solid_sample";

begin
    initial_wait;
    when SATURATION do
        obs_sat(sat_loop, sat_pulse_interval, obs_width_sat, obs_amp_sat, obs_atn);
    end when;

    relaxation_delay;
    obs_width_first,        (obs.gate,      obs.phs.obs_phs_first,   obs.amp.obs_amp_pulse,
    obs.atn.obs_atn);

    loop number_r4 times

    cycle_time_MAS / 4,     (obs.gate,      obs.phs.obs_phs_sr4.lstep(120)      +       90,
    obs.amp.obs_amp_sr4, obs.atn.obs_atn);
    cycle_time_MAS / 4,     (obs.gate,      obs.phs.obs_phs_sr4.lstep(120)      -       90,
    obs.amp.obs_amp_sr4, obs.atn.obs_atn);
    cycle_time_MAS / 4,     (obs.gate,      obs.phs.obs_phs_sr4.lstep(120)      +       90,
    obs.amp.obs_amp_sr4, obs.atn.obs_atn);
    cycle_time_MAS / 4,     (obs.gate,      obs.phs.obs_phs_sr4.lstep(120)      -       90,
    obs.amp.obs_amp_sr4, obs.atn.obs_atn);
    cycle_time_MAS / 4,     (obs.gate,      obs.phs.obs_phs_sr4.lstep(120)      -       90,
    obs.amp.obs_amp_sr4, obs.atn.obs_atn);
```

```
cycle_time_MAS / 4,	(obs.gate,	obs.phs.obs_phs_sr4.lstep(120)	+	90,
obs.amp.obs_amp_sr4, obs.atn.obs_atn);
cycle_time_MAS / 4,	(obs.gate,	obs.phs.obs_phs_sr4.lstep(120)	-	90,
obs.amp.obs_amp_sr4, obs.atn.obs_atn);
cycle_time_MAS / 4,	(obs.gate,	obs.phs.obs_phs_sr4.lstep(120)	+	90,
obs.amp.obs_amp_sr4, obs.atn.obs_atn);

end loop;

parallel begin
	number_saturation/spinning_freq;
justify center
	obs_width_second,	(obs.gate,	obs.phs.obs_phs_second,
obs.amp.obs_amp_pulse,	obs.atn.obs_atn);
justify center
	irr_width_sat,	(irr.gate,	irr.phs.irr_phs_sat,	irr.amp.irr_amp_pulse,
irr.shape.irr_shape_sat, irr.atn.irr_atn);
end parallel;

loop number_r4 times

cycle_time_MAS / 4,	(obs.gate,	obs.phs.obs_phs_sr4.lstep(120)	+	90,
obs.amp.obs_amp_sr4, obs.atn.obs_atn);
cycle_time_MAS / 4,	(obs.gate,	obs.phs.obs_phs_sr4.lstep(120)	-	90,
obs.amp.obs_amp_sr4, obs.atn.obs_atn);
cycle_time_MAS / 4,	(obs.gate,	obs.phs.obs_phs_sr4.lstep(120)	+	90,
obs.amp.obs_amp_sr4, obs.atn.obs_atn);
cycle_time_MAS / 4,	(obs.gate,	obs.phs.obs_phs_sr4.lstep(120)	-	90,
obs.amp.obs_amp_sr4, obs.atn.obs_atn);
cycle_time_MAS / 4,	(obs.gate,	obs.phs.obs_phs_sr4.lstep(120)	-	90,
obs.amp.obs_amp_sr4, obs.atn.obs_atn);
```

```
    cycle_time_MAS / 4,    (obs.gate,    obs.phs.obs_phs_sr4.lstep(120)    +    90,
    obs.amp.obs_amp_sr4, obs.atn.obs_atn);
    cycle_time_MAS / 4,    (obs.gate,    obs.phs.obs_phs_sr4.lstep(120)    -    90,
    obs.amp.obs_amp_sr4, obs.atn.obs_atn);
    cycle_time_MAS / 4,    (obs.gate,    obs.phs.obs_phs_sr4.lstep(120)    +    90,
    obs.amp.obs_amp_sr4, obs.atn.obs_atn);

    end loop;

    acq( dead_time, delay, obs_phs_acq );
end pulse;
```
